# Case report: A rare case of left ventricular noncompaction in two Chinese siblings with becker muscular dystrophy caused by deletion of exons 10 to 12 in the *DMD* gene

**DOI:** 10.3389/fcvm.2023.1243825

**Published:** 2023-09-14

**Authors:** Jingdong Li, Wanyue Zhu, Guanhua Su, Feng Zhu, Xinxin Shuai, Yidi Meng, Jiaming Zhang, Hao Chen

**Affiliations:** ^1^ Department of Cardiology, Union Hospital, Tongji Medical College, Huazhong University of Science and Technology, Wuhan, China; ^2^ Department of Emergency Medicine, Union Hospital, Tongji Medical College, Huazhong University of Science and Technology, Wuhan, China; ^3^ Department of Gerontology, Union Hospital, Tongji Medical College, Huazhong University of Science and Technology, Wuhan, China

**Keywords:** becker muscular dystrophy, left ventricular noncompaction, cardiomyopathy, heart failure, heart transplantation

## Abstract

**Background:**

Becker muscular dystrophy (BMD) is an inherited X-linked recessive condition resulting from mutations of the *DMD* gene encoding dystrophin. Left ventricular noncompaction (LVNC) is a rare cardiomyopathy morphologically characterized by abnormal myocardial trabeculae and deep recesses in the left ventricle. LVNC in BMD patients has only rarely been reported.

**Case report:**

In the present study, we identified a deletion mutation in exons 10 to 12 (EX10_12 del) of the *DMD* gene (reference sequence NM_004006.2) in two Chinese siblings with BMD and LVNC by high throughput targeted next-generation sequencing (NGS) and quantitative polymerase chain reaction (qPCR). The proband was a 22-year-old man admitted with dyspnea, abdominal distention, and polyserositis. It is noteworthy that both the proband and his younger brother manifested progressive muscular atrophy and creatine kinase (CK) elevation. Light and electron microscopy examination of muscle biopsies showed the typical features of dystrophinopathies. Cardiac magnetic resonance imaging and echocardiography demonstrated that both brothers had an enlarged left ventricle, LVNC, and reduced left ventricular ejection fraction. Finally, the proband underwent heart transplantation at age 26 with an event-free follow-up over 4 years post-transplantation.

**Conclusion:**

This case further enriches our knowledge of the symptoms, genotype, cardiac performance, management, and prognosis of BMD patients complicated by LVNC. It is recommended that early comprehensive cardiac evaluation should be considered for patients with BMD to exclude LVNC, as this may have a significant impact on their prognosis.

## 1. Introduction

Muscular dystrophy represents a broad group of hereditary muscle diseases, of which the most prevalent are Duchenne muscular dystrophy (DMD) (OMIM #310200) and Becker muscular dystrophy (BMD) (OMIM #300376). DMD affects about one in 3,500–5,000 newborn males with principal features of slowly progressive muscle weakness in the first few years, loss of ambulation in the second decade, and death from cardiomyopathy or respiratory failure early in the third decade of life (1, 2). In contrast, BMD has an incidence of 1:19,000 and a milder clinical course than DMD (3). Although, cardiac involvement is frequently present in BMD patients (4, 5), left ventricular noncompaction (LVNC) has only rarely been reported. In a study of 186 patients with DMD/BMD, LVNC was found in 19% of the patients. Of those LVNC patients, 32 had DMD while only 3 had BMD (6). Here, we report a rare example of two young adults in a family who presented with both BMD cardiomyopathy and LVNC. They showed the deletion of exons 10 to 12 in the dystrophin gene by next-generation sequencing (NGS) and quantitative polymerase chain reaction (qPCR). Our study may offer fresh perspectives on the pathogenesis, diagnosis, and management of BMD cardiomyopathy complicated with LVNC.

## 2. Case report

In the studied family, the proband, a 22-year-old man, and his 20-year-old brother were born of non-consanguineous parents. Both patients exhibit normal intelligence, devoid of any other neurological symptoms, and no sign of further deterioration to date.

**Figure 1:**
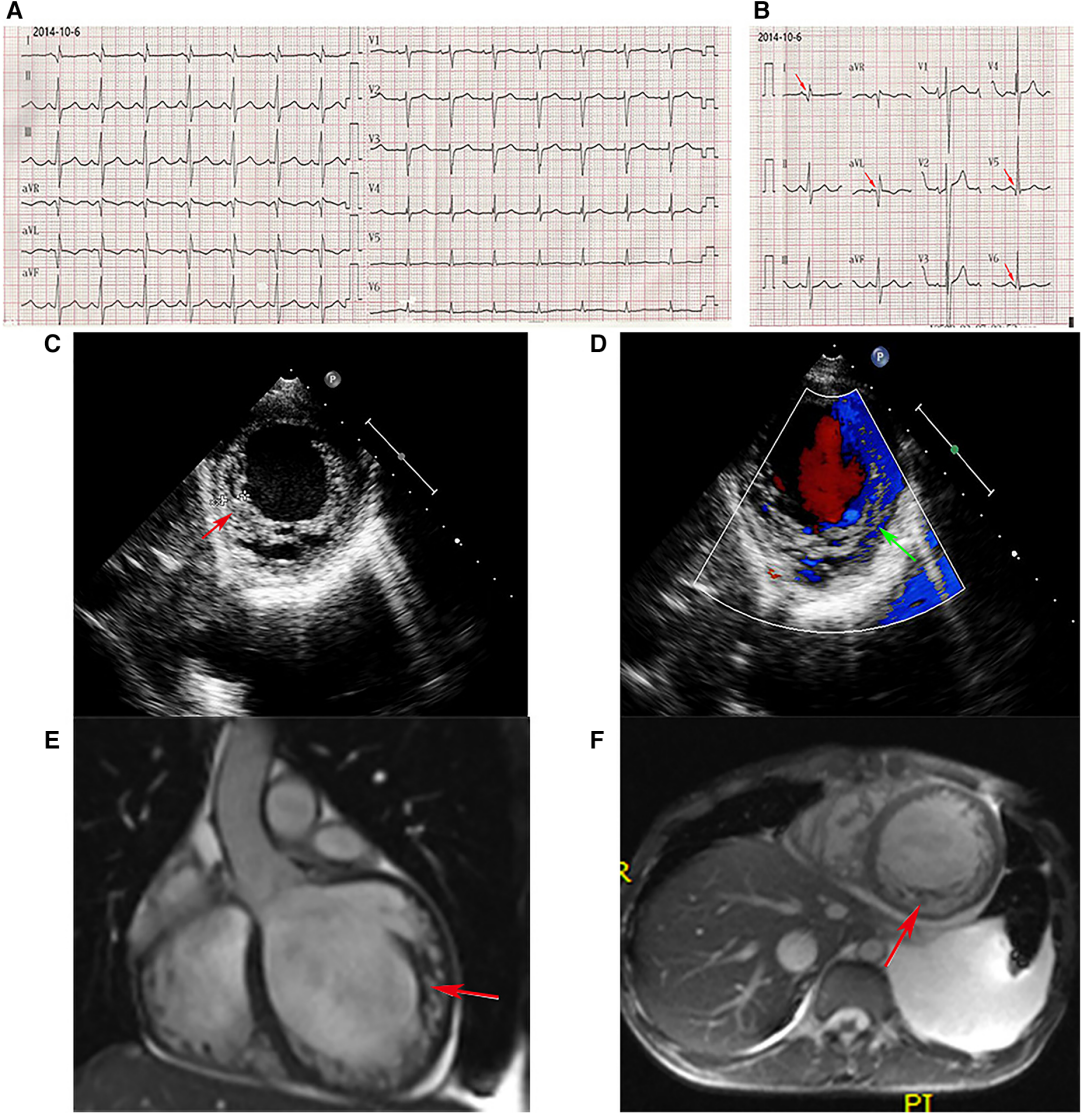
ECG. (**A,B**) Deep and narrow Q waves in leads I, aVL, and V5 to V6 (red arrows). Echocardiography. (**C**) Abundant prominent trabeculations and deep intertrabecular recesses were observed in the left ventricular walls. (**D**) Color flow imaging showed blood flow in intertrabecular recesses. CMR. (**E,F**) The non-enhanced CMR imaging (**E**, coronal position, **F**, short axis) disclosed significant cardiac enlargement, and distinct trabeculations localized to the inferior wall, lateral wall, and part of the anterior wall, as well as the apex.

The proband was admitted to Wuhan Union Hospital because of dyspnea, abdominal distention, and polyserositis, with progressive muscle weakness since the age of 13 years. Biochemical examination showed elevated CK at 11,933 U/L, aspartate transaminase (AST) of 164 U/L, alanine aminotransferase (ALT) of 202 U/L, and N-Terminal Pro-Brain Natriuretic Peptide (NT-pro BNP) of 9,835 pg/ml. Admission electrocardiography (ECG) showed sinus rhythm with a heart rate of 95 beats/min, deep and narrow Q waves in leads I, aVL, and V5 to V6 (Figures 1A,B). The two-dimensional transthoracic echocardiography (TTE) examination revealed prominent trabeculations and deep intertrabecular recesses of the left ventricular (LV) walls and apical areas (Figure 1C). The non-compacted myocardium of the inferior wall apical segment measured 1.8 cm while the compacted myocardium was as thin as 0.4 cm and the systolic non-compacted to compacted ratio was more than 2.0. Echocardiography also demonstrated that the proband had increased LV end-systolic volume (ESV, at 119 ml), LV end-diastolic diameter (LVEDD, of 7.3 cm), and left atrium end-systolic diameter (LAESD, of 4.9 cm) with reduced LV ejection fraction (LVEF, 23%). Notably, color flow imaging showed blood flow in intertrabecular recesses (Figure 1D). Furthermore, the diagnosis of LVNC was verified by cardiac magnetic resonance (CMR). The heart was significantly enlarged on CMR imaging, and distinct trabeculations were localized on the lateral wall, inferior wall, and part of the anterior wall, as well as at the apex (Figures 1E,F). Furthermore, the diastolic non-compacted to compacted ratio was more than 2.3, which was consistent with the diagnosis of LVNC confirmed by TTE.

**Figure 2:**
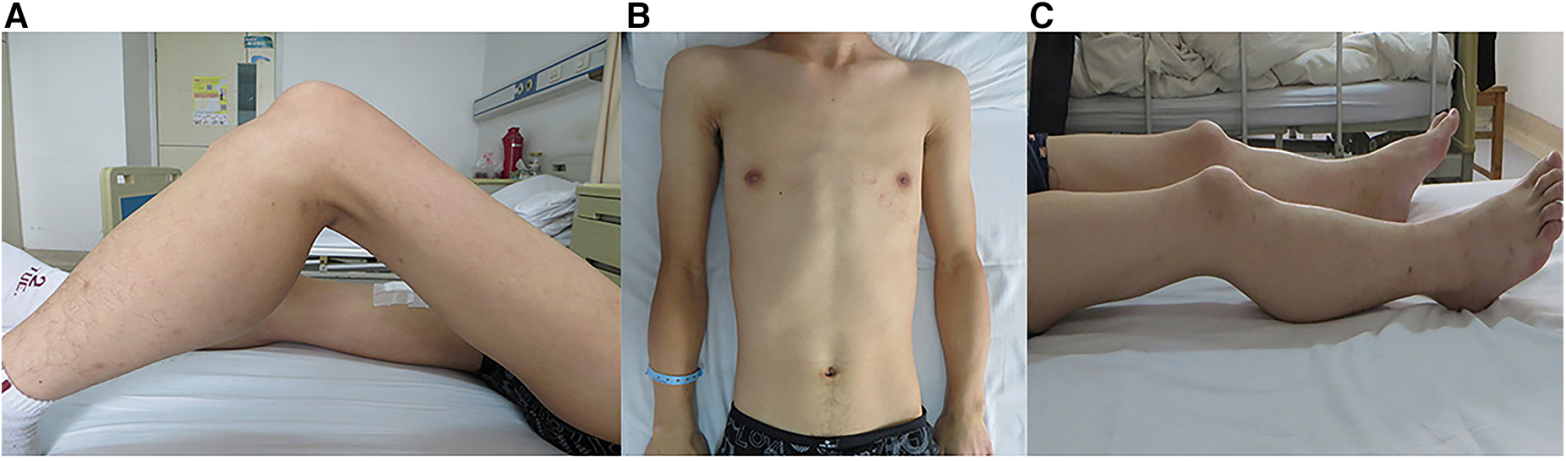
Clinical features in the siblings. Photographs of the proband (**A,B**) and his brother (**C**) showed the clinical features of proximal limb muscle atrophy and calf muscle pseudohypertrophy.

**Figure 3:**
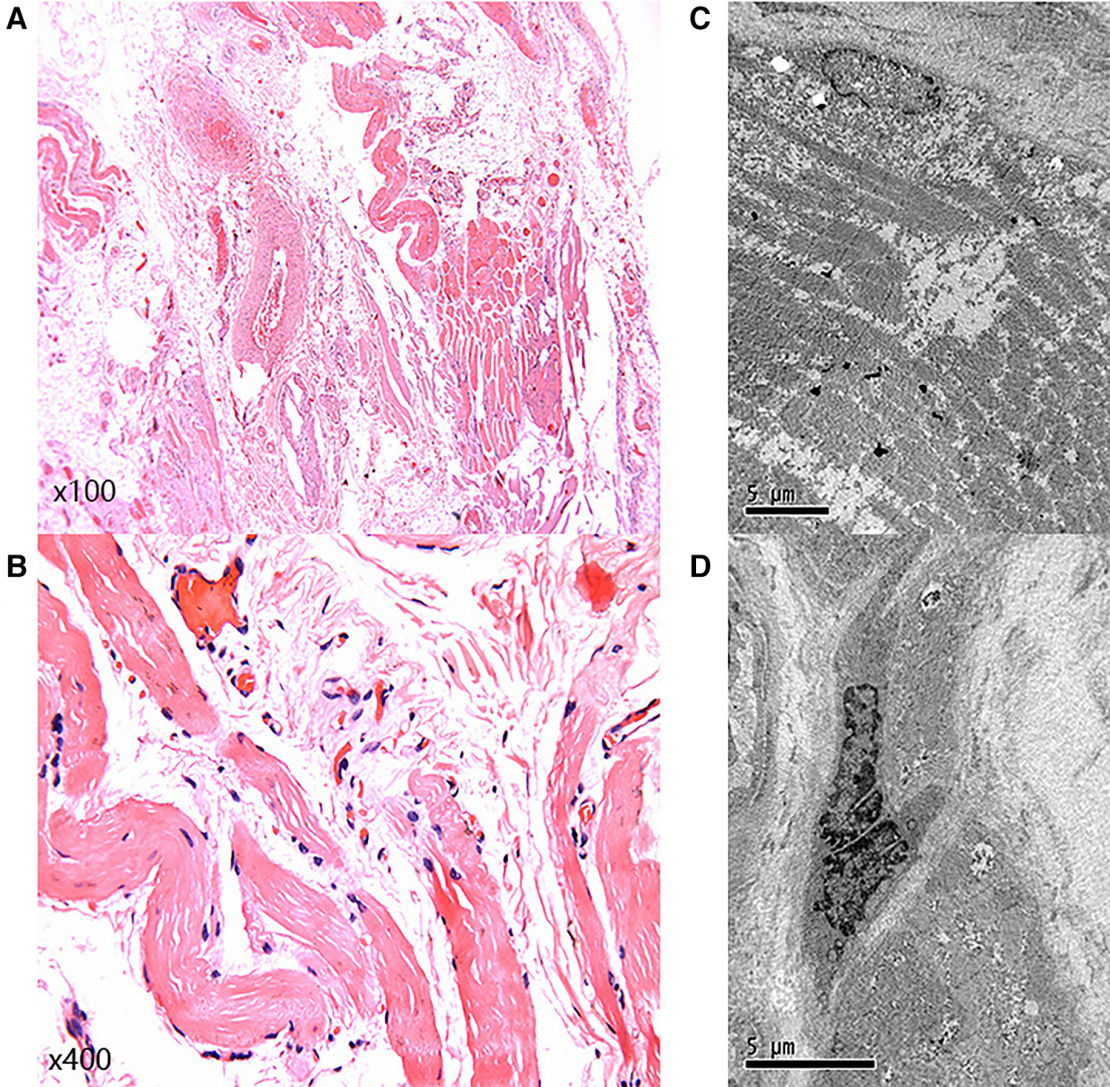
Light microscopy and electron microscopy. (**A,B**) Light microscopy showed variation in muscle fiber size, accompanied by muscle fiber atrophy, hypertrophy of muscle fibers, partial degeneration of muscle fibers, with interstitial fibrosis, and adipose tissue infiltration. (**C,D**) Electron microscopy examination showed swollen mitochondria, expanded sarcoplasmic reticulum, focally dissolved myofilaments, and a few atrophied muscle fibers.

Significantly, the proband showed typical clinical features with progressive proximal limb muscle atrophy and calf muscles pseudohypertrophy (Figures 2A,B). The electromyogram (EMG) of the proband revealed a myogenic lesion. In order to comprehensively evaluate muscle pathology, light microscopy (LM) and electron microscopy (EM) examination were performed. Pathological specimens were obtained from the right quadriceps muscle biopsies of the proband. LM study with hematoxylin-eosin (HE) staining and immunohistochemical (IHC) staining demonstrated variation in muscle fiber size accompanied by atrophy of muscle fibers, hypertrophy of muscle fibers, partial degeneration of muscle fibers with interstitial fibrosis, and adipose tissue infiltration (Figures 3A,B). EM examination showed swollen mitochondria, expanded sarcoplasmic reticulum, focally dissolved myofilaments, and a few atrophied muscle fibers, without abnormal deposits (Figures 3C,D).

Unexpectedly, as the primary caregiver during hospitalization, his younger brother showed proximal limb muscle atrophy and calf muscles pseudohypertrophy similar to the proband (Figure 2C). Based on these findings, further investigations on the younger brother were carried out. Biochemical examination showed elevated CK (3,826 U/L) and TTE indicated LVNC with a dilated left ventricle (6.6 cm) as well as reduced LVEF (25%), although the younger brother did not complain of any cardiac symptoms.

**Figure 4:**
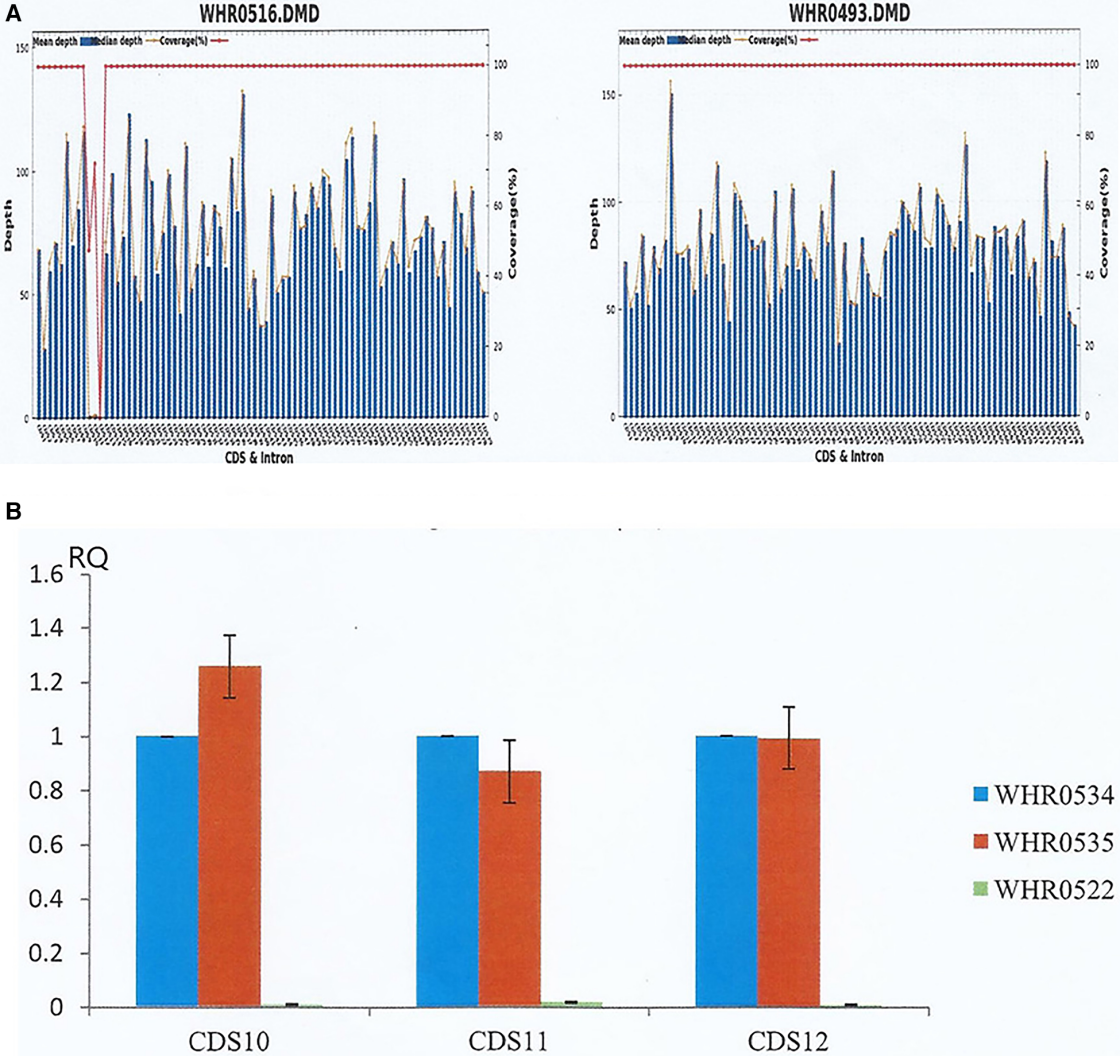
NGS and qPCR results of the siblings. (**A**) The proband (left, WHR0516) and a normal male (right, WHR0493). NGS determined the average sequencing depth and coverage of the target area in *DMD* gene, revealing deletion of exons 10–12 deletion (EX10_12 del) in the *DMD* gene of the proband. (**B**) WHR0534 and WHR0535 are two normal controls, while WHR0522 represented the proband's brother. The coding sequence of the *DMD* gene from the proband's brother was verified by qPCR, confirming the deletion of the same exons as the proband.

Consequently, we suspected that both siblings suffered from hereditary muscular dystrophy and familial dilated cardiomyopathy. To confirm the diagnosis, we performed high throughput targeted NGS analysis on genomic DNA samples obtained from the proband with a customized panel encompassing 29 genes involved in the pathogenesis of hereditary muscular dystrophy. The DNA library was prepared using the KAPA Hyper Prep Kit (KAPA Biosystems), followed by target enrichment based on a probe library (Roche NimbleGen SeqCap EZ Choice). Finally, the products were analyzed by NGS using the Illumina platform, MiSeq. Results indicated a deletion of exons 10–12 (EX10_12 del) in the *DMD* gene (reference sequence NM_004006.2) (Figure 4A), affecting the normal expression of dystrophin. Moreover, we confirmed the deletion of the same exons in the *DMD* gene in his younger brother by qPCR (Figure 4B).

After discharge from the hospital, the proband was on long-term treatment with a β-blocker, a diuretic, coenzyme Q10, and an angiotensin-converting enzyme inhibitor (ACEI). However, he required repeated hospitalization for heart failure, with gradually worsening muscle weakness, over the following 4 years. Finally, at the age of 26, he underwent orthotopic heart transplantation and has remained in good condition since transplantation.

## 3. Discussion

DMD/BMD should be suspected when there are characteristic clinical features; an increased CK, and a family history of muscular dystrophy. To confirm the diagnosis, genetic testing is required (7). Both DMD and BMD are X-linked recessive conditions resulting from mutations in the *DMD* gene located at Xp21.2 (8). The *DMD* gene encodes the protein dystrophin, which is a major intermediate protein of the myocardium and skeletal muscle. DMD results from out-of-frame mutations that shift the mRNA's reading frame of dystrophin, causing paucity or absence of muscle dystrophin. By contrast, the milder BMD is caused by in-frame mutations maintaining the reading frame of the *DMD* gene, causing the synthesis of partially functioning dystrophin (9, 10). In our patients, the siblings, who carried an in-frame deletion, could walk independently without wheelchairs in their 20s and showed few atrophied muscle fibers on pathological examination. Based on these findings, the diagnosis of BMD was established.

Myocardial involvement is a frequent feature in BMD. More than 70% of patients will develop cardiomyopathy, and this is the leading cause of death in BMD. Symptomatic myocardial involvement usually occurs in the third decade. Patients with BMD have a relatively milder skeletal muscle phenotype compared to those with DMD, but cardiomyopathy may be more severe (3–5). Patients with BMD may experience a variety of cardiac phenotypes, the most common of which include dilated cardiomyopathy (DCM), heart failure, hypertrophic cardiomyopathy, arrhythmias, and cardiac arrest (11). In the present study, we found that the myocardial involvement manifested as LVNC, which is a relatively rare finding in patients with BMD.

LVNC is a rare cardiomyopathy morphologically characterized by prominent trabeculations, deep intertrabecular recesses, and a thin compacted layer in the LV walls. It is still debated whether LVNC represents a distinct cardiomyopathy or may be a morphologic characteristic shared by various cardiomyopathies, such as Barth syndrome and DMD/BMD. LVNC is genetically heterogeneous. Human genetic studies suggest that LVNC is associated with mutations in a variety of genes, including myosin heavy chain 7 (MYH7), myosin binding protein C3 (MYBPC3), LIM domain binding protein 3 (ZASP), tafazzin (TAZ/G4.5), desmin (DES), and others (12–16). 20%–40% of LVNC cases tend to be familial, with inheritance as an autosomal dominant or X-linked recessive disorder. Clinical manifestations are highly diverse in LVNC patients, even in the same family (12, 17). LVNC was first described in a BMD patient approximately 30 years ago: CMR revealed abnormal trabeculation in the LV apex and nearby lateral wall of a 33-year-old male with BMD caused by a deletion of exons 45 to 48 in the *DMD* gene (18). It was confirmed, in a subsequent report, that this patient died at the age of 40 years from intractable heart failure (19). In 2001, Finsterer et al. reported a case of spontaneous LVNC in a BMD patient resulting from a duplication in the *DMD* gene (20). In the subsequent two decades, there were no further reports of LVNC in BMD patients until 2021. In 2021, Shah et al. reported a case of a 50-year-old man with BMD caused by the deletion of exons 45 to 53 in the *DMD* gene. LVNC was identified through TTE and CMR. Eventually, due to recurrent malignant arrhythmias and syncope, he underwent implantation of an implantable cardioverter-defibrillator (ICD) and LV assist device, as well as preparation for heart transplantation (21). In DMD/BMD, LVNC appears to exacerbate the deterioration of LV function, leading to higher mortality (6, 22, 23).

To the best of our knowledge, this is the first case report of heart transplantation in a BMD patient with LVNC. With advances in the management of respiratory failure, heart disease has become the major cause of death in patients with muscular dystrophy (24). The principles of treatment of dystrophic cardiomyopathy are based on early diagnosis, and include alleviation of symptoms and inhibition of heart disease progression. Currently, therapy, in dystrophic cardiomyopathies, includes small molecule-based treatments which have been shown to be beneficial, such as corticosteroids, ACEIs, mineralocorticoid receptor antagonists, and beta-adrenergic receptor blockers. Gene-targeted therapies for DMD/BMD, such as stop codon readthrough, exon skipping, and CRISPR/Cas9 (clustered regularly interspaced short palindromic repeats/CRISPR-associated protein 9) gene editing, also bring great opportunities and challenges (25, 26). However, for DMD/BMD patients with end-stage heart failure, without other life-threatening comorbidities, orthotopic heart transplantation should be considered, as in our proband patient (3, 27).

To date, there are no known mouse or cell culture models adequately reflecting the genotype. For decades the C57BL/10ScSn-Dmdmdx/J mouse has been the most commonly used model of DMD. However, BMD mouse models are rare. Previous studies have shown the feasibility of generating a DMD/BMD mouse model by exon deletion using CRISPR/Cas9 gene editing techniques (28, 29). For example, Christopher R et al. used this technology to generate Bmx (Becker muscular dystrophy, X-linked) mice, which express an in-frame deletion of exons 45 to 47 in the murine *DMD* gene (29). These studies provide new approaches and further research will generate novel data and concepts.

In conclusion, our 2 patients have provided new insights into the diagnosis, pathophysiology, genetic mechanism, and treatment of BMD patients with LVNC. We suggest that patients with BMD should be considered for early comprehensive cardiac evaluation to exclude LVNC, with the aim of preventing the development of severe end-stage heart failure.

## Data Availability

The raw data supporting the conclusions of this article will be made available by the authors, without undue reservation.
